# Extensive Recombination-driven Coronavirus Diversification Expands the Pool of Potential Pandemic Pathogens

**DOI:** 10.1093/gbe/evac161

**Published:** 2022-11-04

**Authors:** Stephen A Goldstein, Joe Brown, Brent S Pedersen, Aaron R Quinlan, Nels C Elde

**Affiliations:** Department of Human Genetics, University of Utah, Salt Lake City, Utah, USA; Howard Hughes Medical Institute, Chevy Chase, Maryland, USA; Department of Human Genetics, University of Utah, Salt Lake City, Utah, USA; Department of Human Genetics, University of Utah, Salt Lake City, Utah, USA; Department of Human Genetics, University of Utah, Salt Lake City, Utah, USA; Department of Human Genetics, University of Utah, Salt Lake City, Utah, USA; Howard Hughes Medical Institute, Chevy Chase, Maryland, USA

**Keywords:** virology, evolution, coronaviruses

## Abstract

The ongoing SARS-CoV-2 pandemic is the third zoonotic coronavirus identified in the last 20 years. Enzootic and epizootic coronaviruses of diverse lineages also pose a significant threat to livestock, as most recently observed for virulent strains of porcine epidemic diarrhea virus (PEDV) and swine acute diarrhea-associated coronavirus (SADS-CoV). Unique to RNA viruses, coronaviruses encode a proofreading exonuclease (ExoN) that lowers point mutation rates to increase the viability of large RNA virus genomes, which comes with the cost of limiting virus adaptation via point mutation. This limitation can be overcome by high rates of recombination that facilitate rapid increases in genetic diversification. To compare the dynamics of recombination between related sequences, we developed an open-source computational workflow (IDPlot) that bundles nucleotide identity, recombination, and phylogenetic analysis into a single pipeline. We analyzed recombination dynamics among three groups of coronaviruses with noteworthy impacts on human health and agriculture: *SARSr-CoV*, *Betacoronavirus-1*, and SADSr-CoV. We found that all three groups undergo recombination with highly diverged viruses from undersampled or unsampled lineages, including in typically highly conserved regions of the genome. In several cases, no parental origin of recombinant regions could be found in genetic databases, demonstrating our shallow characterization of coronavirus diversity and expanding the genetic pool that may contribute to future zoonotic events. Our results also illustrate the limitations of current sampling approaches for anticipating zoonotic threats to human and animal health.

**Significance**

Most focus on RNA virus evolution concerns the role of point mutation, but recombination plays an underappreciated role in generating coronavirus genetic diversity. We describe recombination between highly divergent viruses, demonstrating that characterized viruses occupy an overlapping niche, and recombine with yet-undiscovered viral lineages. Current surveillance approaches are ill-equipped to comprehensively identify nascent threats to public health.

## Introduction

In the 21st century alone three zoonotic coronaviruses have caused widespread human infection: SARS-CoV in 2002 (Drosten et al. 2003; Peiris et al. 2003), MERS-CoV in 2012 (Zaki et al. 2012), and SARS-CoV-2 in 2019 (Zhou, Yang, et al. 2020). Four other coronaviruses, OC43, 229E, NL63, and HKU1 are endemic in humans and cause mild-to-moderate respiratory disease with low fatality rates, though they may cause outbreaks of severe disease in vulnerable populations (Patrick et al. 2006; Hand et al. 2018; Killerby et al. 2018; Zeng et al. 2018). Like SARS-CoV-2, SARS-CoV, and MERS-CoV, these endemic viruses emerged from animal reservoirs. The origins of 229E and NL63 have been convincingly linked to bats, much like the 21st-century novel coronaviruses (Pfefferle et al. 2009; Huynh et al. 2012; Tao et al. 2017). In a striking parallel, both MERS-CoV and 229E appear to have emerged from bats into camelids, established a new persistent reservoir, and then spilled over into humans (Crossley et al. 2012; Corman et al. 2014, 2016; Khalafalla et al. 2015). In contrast, the viral lineages that include OC43 and HKU1 originated in rodents (Lau, Woo, et al. 2015; Wang et al. 2015), though the deep evolutionary history of these viruses remains mysterious. Given the short infectious period of human coronavirus infections, the establishment of endemicity was likely preceded by a period of intense and widespread transmission on regional or global scales. In other words, SARS-CoV-2 is likely the fifth coronavirus epidemic or pandemic involving a still-circulating virus, though the severity of the previous four is unknown. We further cannot discount the possibility of past coronavirus epidemics caused by now-extinct viruses.

Livestock is similarly impacted by the spillover of coronaviruses from wildlife reservoirs. Three viruses closely related to OC43, bovine coronavirus (BCoV), equine coronavirus (EcoV), and porcine hemagglutinating encephalomyelitis virus (PHEV) are enzootic or epizootic in cows, horses, and pigs, respectively (Vijgen et al. 2006; Zhang et al. 2007). Since 2017, newly emerged swine acute diarrhea syndrome-associated coronavirus (SADS-CoV) has caused significant mortality of piglets over the course of several outbreaks (Gong et al. 2017; Li et al. 2018). A sampling of bats proximal to impacted farms determined that SADS-CoV outbreaks are independent spillover events of SADSr(elated)-CoVs circulating in horseshoe bats (Zhou et al. 2018). Molecular studies of a prototypical SADS-CoV have identified the potential for further cross-species transmission, including the ability to infect primary human airway and intestinal cells (Yang et al. 2019; Edwards et al. 2020).

Emergence of novel viruses requires access to new hosts, often via ecological disruption, and the ability to efficiently infect these hosts, frequently driven by adaptive evolution. Uniquely among RNA viruses, coronavirus genomes encode a proofreading exonuclease that results in a significantly lower mutation rate for coronaviruses compared to other RNA viruses (Eckerle et al. 2007). This mutational constraint is necessary for maintaining the stability of the large (27–32 kilobases (kb)) RNA genome but limits the evolution of coronaviruses via point mutation. The high recombination rate of coronaviruses compensates for the adaptive constraints imposed by high-fidelity genome replication (Smith and Denison 2012). The spike glycoprotein in particular has previously been identified as a recombination hotspot (Graham and Baric 2010). Acquisition of new spikes may broaden or alter receptor usage, enabling host-switches or expansion of host range. Additionally, it may result in the evasion of population immunity within established host species, effectively replenishing the pool of susceptible individuals. Recombination in other regions of the genome is less well-documented but may also influence host range, virulence, and tissue tropism, and likely contributed to the emergence of SARS-CoV (Yang et al. 2016; Hu et al. 2017).

To study the dynamics of recombination among clinically significant coronavirus lineages we developed a novel web-based software, IDPlot, that incorporates multiple analysis steps into a single user-friendly workflow. Analyses performed by IDPlot include multiple sequence alignment, nucleotide identity analysis, and tree-based breakpoint prediction using the GARD algorithm from the HyPhy genetic analysis suite (Kosakovsky Pond et al. 2006). IDPlot also allows the direct export of sequence regions to NCBI Blast to ease the identification of closest relatives to recombinant regions of interest. The IDPlot output is uniquely interactive and user-friendly, in particular, easing analysis of GARD results through an interactive display of iterative model fit improvement.

We used IDPlot to analyze the recombination history of three clinically significant lineages of coronaviruses: SARS-CoV-2-like viruses and OC43-like viruses (*Betacoronavirus-1*) in the *Betacoronavirus* genus, and the SADSr-CoV group of alphacoronaviruses. In all three groups, we found clear evidence of recombination resulting in viruses with high overall nucleotide identity but exhibiting substantial genetic divergence in discrete genomic regions. Recombination was particularly enriched around and within the spike gene and 3′ accessory genes but major recombination events also encompassed the normally conserved, evolutionarily constrained Orf1ab and S2 domain of the spike. These latter findings informed a critical element of our analyses; whether extended branch lengths and changes in phylogenetic tree topologies were due to rapid evolution versus recombination. Significant decreases in nucleotide identity in conserved regions such as Orf1ab and S2 are strong evidence of recombination. For putative recombination events involving only variable regions of the genome (such as spike S1) rapid evolution may be more difficult to exclude, though the degree of divergence, even accounting for rapid evolution, was generally discordant with the short evolutionary distance between related viruses in the rest of their genomes.

Within all three groups, recombination has occurred with distant under- or unsampled lineages, but our most striking findings were with respect to SADSr-CoVs in which we identified six unique spikes and five Orf7a genes within an eight-virus dataset. These viruses are emerging livestock pathogens at risk of human emergence. We identified major recombination events in this lineage generating diversity among otherwise highly similar viruses, indicative of frequent recombination between distantly related viruses. The potential for viruses to acquire novel phenotypes through such recombination events underscores the importance of robust and coordinated ecological, public health, and research responses to the pandemic threat of coronaviruses.

## Results

### Coronavirus Phylogenetic Relatedness is Variable Across Genomes

**Fig. 1.**
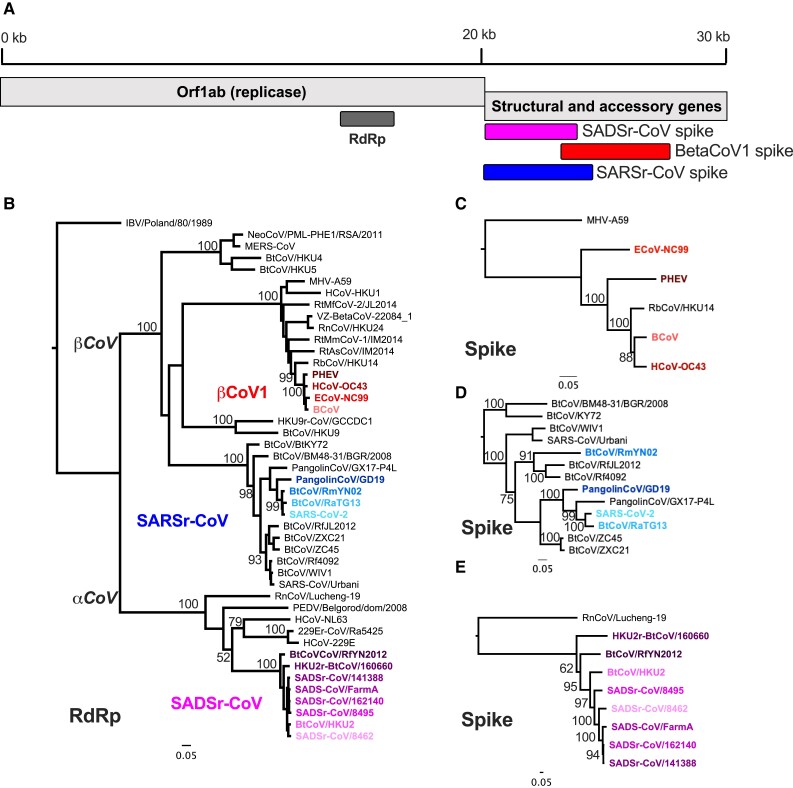
AlphaCoV and BetaCoV phylogenetic relationships are genome region-dependent. (*A*) Basic coronavirus genome organization with the 5′ ∼20 kb comprising the replicase gene that is proteolytically processed into up to 16 individual proteins. The 3′ 10 kb comprises structural and genus-specific accessory genes. (*B*) Maximum-likelihood (ML) phylogenetic tree of alpha and betaCoVs full-length RNA-dependent RNA-polymerase encoding region of Orf1ab. (*C*) ML phylogenetic tree of full-length spike genes from viruses in the species *Betacoronavirus 1* (red) rooted with the distantly related betacoronavirus mouse hepatitis virus. (*D*) ML phylogenetic tree of spike genes of SARSr-CoVs, with SARS-CoV-2-like viruses further analyzed in the paper highlighted in blue. (*E*) ML phylogenetic tree of spike genes from SADSr-CoVs (magenta) rooted with the distantly related alphacoronavirus RnCov/Lucheng-19.

Coronavirus genomes, at 27–32 kb in length, are among the largest known RNA genomes, surpassed only by invertebrate viruses in the same *Nidovirales* order (Debat 2018; Saberi et al. 2018). The 5′ ∼20 kb of the genome comprises open reading frames 1a and 1b, which are translated directly from the genome as polyproteins pp1a and pp1ab and proteolytically cleaved into constituent proteins (fig. 1*A*) (Fehr and Perlman 2015). Orf1ab is among the most conserved genes and encodes proteins essential for replication, including the RNA-dependent RNA-polymerase (RdRp), 3C-like protease (3ClPro), helicase, and methyltransferase. Given the high degree of conservation in this region, coronavirus species classification is typically determined by the relatedness of these key protein-coding regions (Group 2020). The 3′ ∼10 kb of the genome contains structural genes including those encoding the spike and the nucleocapsid proteins, as well as accessory genes (numbered ORFs) that are unique to coronavirus genera and subgenera (Liu et al. 2014). The structural and accessory region, and in particular the spike glycoprotein gene, has been identified as a recombination hotspot.

We set out to characterize the role of recombination in generating diversity across the coronavirus phylogeny. A classic signature of recombination is different phylogenetic tree topology depending on what genomic regions are analyzed, while in undersampled lineages extended branch lengths may reflect recombination rather than rapid mutational divergence. To identify lineages of interest for recombination analysis, we built a maximum-likelihood phylogenetic tree of full-length RdRp-encoding regions of representative alpha and betacoronaviruses, which contain all human and most mammalian coronaviruses (fig. 1*B*). To further test whether comparisons of RdRp sequence reflected ancestral relatedness, we conducted the same analysis for the 3ClPro and Helicase-encoding regions of Orf1ab (supplementary fig. S2, Supplementary Material online). Phylogenetic relationships were generally maintained in these trees and genetic relatedness remains very high (90–99% within groups), so any shuffling of phylogenetic relationships between these regions had low bootstrap support. From these trees we chose to further investigate the evolutionary dynamics of three clinically significant groups of coronaviruses: Four SARS-CoV-2 like viruses (blue) from within *SARSr(elated)-CoV* as a test-run of our novel workflow, four endemic and enzootic OC43-like viruses of *Betacoronavirus-1* (*BetaCoV1*) (red), and eight SADSr-CoVs (magenta). Although other coronavirus lineages are of public health interest, such as those including the human coronaviruses HKU1, NL63, and 229E there is a relative paucity of closely related sequences to these viruses, limiting our current ability to analyze the evolutionary history of these virus genomes.

Within each of the three selected groups group there is little diversity revealed by comparing RdRp sequence: 91–99% among the SARS-CoV-2-like viruses, > 97% nucleotide (nt) identity within *Betacoronavirus-1*, and 94–99% nt identity among the SADSr-CoVs (supplementary fig. S3, Supplementary Material online). Similar results were observed for 3ClPro and Helicase-encoding regions (supplementary fig. S4, Supplementary Material online). In contrast, spike gene phylogenetic trees of each group show greater diversity as illustrated by extended branch lengths and/or changes in tree topology, suggesting rapid evolution and/or recombination-driven diversification in this region (fig. 1*B*–*D*). To analyze these two possibilities and conduct efficient, robust analyses, we developed a new pipeline to better study these evolutionary patterns.

### IDPlot Facilitates Nucleotide Identity and Recombination Analysis

To investigate possible recombination-driven diversity among these viruses we developed IDPlot, which incorporates several distinct analysis steps into a single Nextflow workflow (Di Tommaso et al. 2017) and generates a comprehensive HTML report to facilitate interpretation and downstream analysis. IDPlot combines the existing algorithms MAFFT, GARD, and FastTree2 into a single pipeline along with an innovative, interactive user display to produce a significant advance in nucleotide identity and recombination analysis. Future updates to IDPlot will include a menu of recombination detection programs suited to different datasets and computational capacities.

**Fig. 2.**
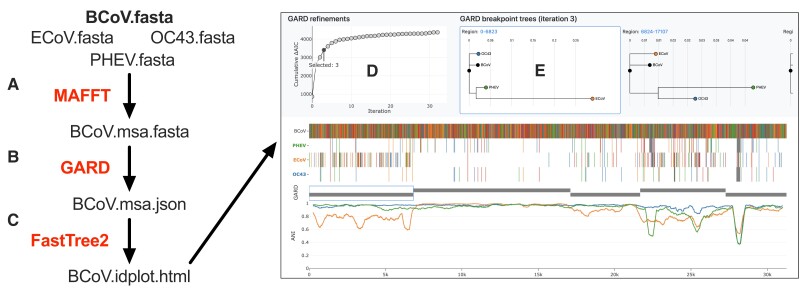
IDPlot workflow. (*A*) Reference and query sequences are aligned using MAFFT. (*B*) Breakpoint detection is performed using GARD, capturing breakpoints across iterative refinements. (*C*) Phylogenetic trees based on breakpoints from each iteration and are created using FastTree 2. (*D*) Improvement in ΔAIC-c is plotted against the iteration. (*E*) Phylogenetic trees associated with the selected GARD iteration are displayed.

IDPlot runs its constituent algorithms sequentially to produce a recombination report. First, IDPlot generates a multiple sequence alignment using MAFFT (fig. 2*A*) (Katoh 2002) with user-assigned reference and query sequences, though IDPlot will also accept a custom alignment. In its default configuration, IDPlot then generates a 500-nt sliding window average nucleotide identity (ANI) plot and displays the multiple sequence alignment above the ANI plot with differences to the reference sequence (colored vertical lines) and gaps (gray boxes) clearly highlighted. The plot is zoomable, and selected sequence regions can be exported directly to NCBI BLAST. Users can also choose to run GARD, the recombination detection program from the HyPhy suite of genomic analysis tools (Kosakovsky Pond et al. 2006). If GARD is implemented (fig. 2*B*), distinct regions of the multiple sequence alignment between breakpoints are depicted between the alignment and the ANI plot, and phylogenetic trees for each region are generated using FastTree2 (fig. 2*C*) (Price et al. 2010) and displayed (fig. 2*E*).

We chose the GARD tool to propose recombination sites in IDPlot due its use of phylogenetic inference, providing breakpoint detection that does not depend on the presence of parental genomes in the data set. GARD can detect recombination in the data set with both sampled and uncharacterized viral lineages, providing the ability to explore recombination with newly discovered virus sequences. However, a significant barrier to effective use of GARD is that because it ultimately presents multiple (sometimes dozens of) iterations that increasingly include spurious breakpoints with marginally improved statistical support, the model choice is difficult and requires qualitative assessment. To resolve this issue the IDPlot output includes a graph showing a cumulative count of GARD's statistical metric Akaike information criterion (AIC-c) on the *y*-axis and the iteration on the *x*-axis (fig. 2*D*). GARD uses ΔAIC-c for each proposed model to indicate the degree of fit improvement over the preceding model, and this graph allows the user to easily determine when improvements become increasingly marginal, indicating that new breakpoints lack strong statistical support. Upon selection of a GARD iteration, the display switches to show the associated phylogenetic trees (fig. 2*E*). Genomic regions are clickable, immediately bringing the appropriate phylogenetic tree to the center of the display. Finally, the ability to export sequences directly to BLAST enables the user to search for related sequences in GenBank, useful when defined regions are highly divergent from the reference sequence and others included in the data set.

### SARS-CoV-2-like Virus Recombination With Distant SARSr-CoVs

To test IDPlot as a tool for examining the recombination dynamics of coronaviruses, we initially conducted an analysis of SARS-CoV-2-like viruses within *SARSr-CoV*. We chose these viruses as our initial IDPlot case study due to their early availability and because recombination had been previously described (Boni et al. 2020), providing the opportunity to evaluate IDPlot against a known framework but also advance our understanding of the role recombination has played in the evolution of these clinically significant viruses.

Prior to 2019 the SARS-CoV-2 branch within *SARSr-CoV* was known only from a single, partial RdRp sequence published in 2016 (Ge et al. 2016) now classified as the closely related virus RaTG13, as well as the distantly related bat viruses ZC45 and ZXC21 (Hu et al. 2018). Additional representatives from bats and pangolins have since been identified (Lam et al. 2020; Murakami et al. 2020; Wahba et al. 2020; Xiao et al. 2020; Zhou, Chen, et al. 2020; Hul et al. 2021; Li et al. 2021; Temmam et al. 2021; Wacharapluesadee et al. 2021; Zhou et al. 2021). Although we primarily selected these viruses as an IDPlot validation panel, we saw the opportunity to add phylogenetic detail to our understanding of their evolutionary history. Most attention on these viruses to date has focused on the recent evolutionary history of SARS-CoV-2 (Boni et al. 2020) or adopted a bigger-picture perspective (Lytras et al. 2021). Less attention has been paid to analyzing the evolution of known close relatives, including the earliest identified closely related viruses, bat viruses RaTG13 and RmYN02, and PangolinCoV/GD19.

**Fig. 3.**
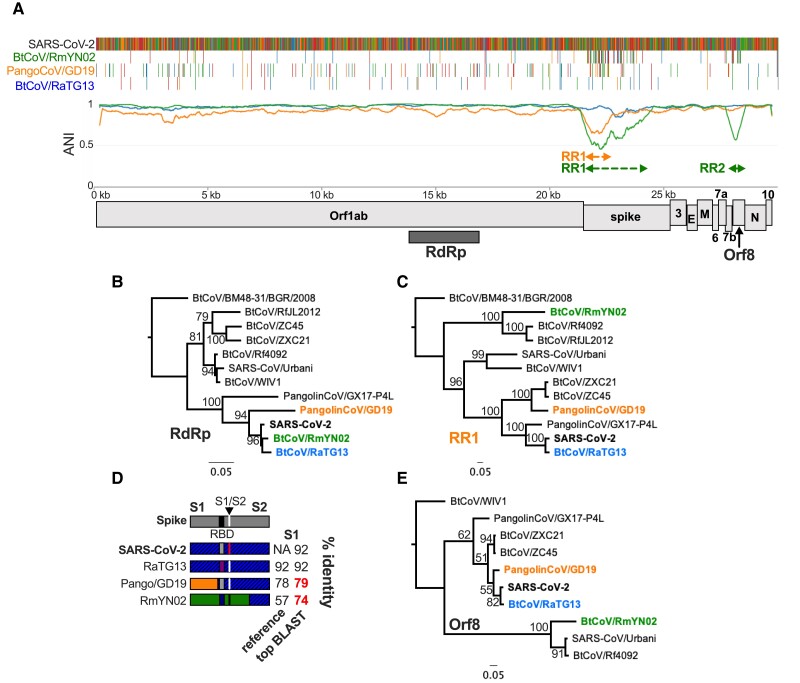
SARSr-CoV IDPlot analysis. (*A*) IDPlot analysis of SARS-CoV-2-like SARSr-CoVs with color-coded dashed lines defining divergent regions arising from recombination events with ancestral viruses. (*B*) ML tree of the RdRp-encoding region of SARS-2-like and other SARSr-CoVs showing close relationship between the SARS-CoV-2-like viruses. (*C*) ML tree of PangolinCoV/GD19 RR1 (which overlaps with BtCoV/RmYN02 RR1) showing different topology than the RdRp tree. (*D*) Schematic of spike proteins indicating divergent regions and nucleotide identity to the reference sequence and closest related sequence in GenBank. (*E*) ML tree of ORf8 showing that RmYN02 Orf8 is a divergent member of the SARS-CoV-like Orf8 branch.

Our IDPlot analysis does not support an emergence of SARS-CoV-2 via recent recombination, consistent with previously published work (Boni et al. 2020). RaTG13 shows consistently high identity across the genome with the only notable dip comprising the receptor-binding domain in the C-terminal region of spike S1 (fig. 3*A*), which is proposed to have been acquired via recombination (Boni et al. 2020). However, the still limited sampling in the SARS-CoV-2-like lineage results in weak phylogenetic signals for the RaTG13 receptor-binding domain.

In contrast, PangolinCoV/GD19 and RmYN02 show one and two significant drops in ANI, respectively. Phylogenetic analysis of the PangolinCoV/GD19 recombinant region captures the signal for both that virus (fig. 3*A* and *C*, supplementary fig. S5*C*, Supplementary Material online) and RmYN02 Recombinant Region 1 (RR1), showing, in agreement that both viruses fall onto separate branches highly divergent from SARS-CoV-2 and RaTG13 (fig. 3*C*) with only 81 and 74% of nucleotide identity to the closest sequences in GenBank, respectively (fig. 3*D*, supplementary fig. S5*A*, Supplementary Material online). These findings identify three unique spike genes among these four viruses (fig. 3*D*), indicative of recombination with undiscovered *SARSr-CoV* lineages, despite being the focus of intense virus sampling efforts over the last eighteen years since the emergence of SARS-CoV.

In addition to spike, RmYN02 contains a second recombinant region that encompasses the 3′ end of Orf7b and the large majority of Orf8 (fig. 3*A*, supplementary fig. S5*A*, Supplementary Material online). Orf8 is known to be highly dynamic in SARSr-CoVs. SARS-CoV underwent an attenuating 29-nt deletion in Orf8 in 2002–2003 (Muth et al. 2018) and Orf8 deletions have been identified in numerous SARS-CoV-2 isolates as well (Pereira 2020; Su et al. 2020; Young et al. 2020). In bat SARSr-CoVs intact Orf8 is typically though not always present but exhibits a high degree of phylogenetic incongruence with the rest of the genome. Additionally, the progenitor of SARS-CoV encoded an Orf8 gene gained via recombination (Lau, Feng, et al. 2015; Hu et al. 2017). The BtCoV/RmYN02 Orf8 has only 50% nt identity to SARS-CoV-2 Orf8 and groups as a distantly related member of the branch containing SARS-CoV (fig. 3*E*), providing evidence of recombination between SARS-CoV and SARS-CoV-2-like viruses. Although the precise function of Orf8 is unknown, there is some evidence that like other accessory proteins it mediates immune evasion [43]. Therefore, recombination in Orf8 has the potential to alter virus–host interactions and may, like spike recombination, impact host range and virulence.

This analysis, particularly with respect to RaTG13 and PangolinCoV/GD19 generated results in agreement with prior work (Boni et al. 2020), confirming that IDPlot allows us to robustly characterize recombination events in detail with a single workflow. By extending our analysis to BLAST, facilitated by direct export from IDPlot, we demonstrate that multiple SARS-CoV-2-like viruses have recombined with unsampled SARSr-CoV lineages, showing the utility of IDPlot in conducting more thorough evolutionary analyses than previously available in a single pipeline.

### OC43-like Viruses Encode Divergent Spikes Acquired From Unsampled Betacoronaviruses

After validating IDPlot for recombination analysis of coronaviruses, we used it to characterize recombination among the viruses in the *Betacoronavirus-1* (*BetaCov1*) group, which includes the human endemic coronavirus OC43 and closely related livestock pathogens BCoV, equine coronavirus (ECoV), PHEV, and Dromedary camel coronavirus HKU23 (HKU23). Due to the apparent low virulence of OC43 and limited sampling of the lineage, these viruses receive little attention outside agricultural research. However, this lineage has produced a highly transmissible human virus that can cause severe disease in vulnerable adults, contains several known livestock pathogens, and is poorly sampled. An ancestral BCoV is inferred to be the progenitor of extant *BetaCoV1* viruses with divergence dates estimated at 100–150 years ago for OC43/PHEV (Vijgen et al. 2006) and 50 years ago for HKU23 (Woo et al. 2014). ECoV was originally characterized as the earliest diverging member of the group (Zhang et al. 2007) based on its lowest genome-wide identity to BCoV. Recombination with other betacoronaviruses has been previously described in detail for HKU23, so we excluded it from our analysis (So et al. 2019). The most closely related known virus to *BetaCoV1*, rabbit coronavirus HKU14 (RbCoV/HKU14) was reported to associate with ECoV in some regions (Lau et al. 2012), but no detailed recombination analysis of the relationship between these viruses has been previously described.

**Fig. 4.**
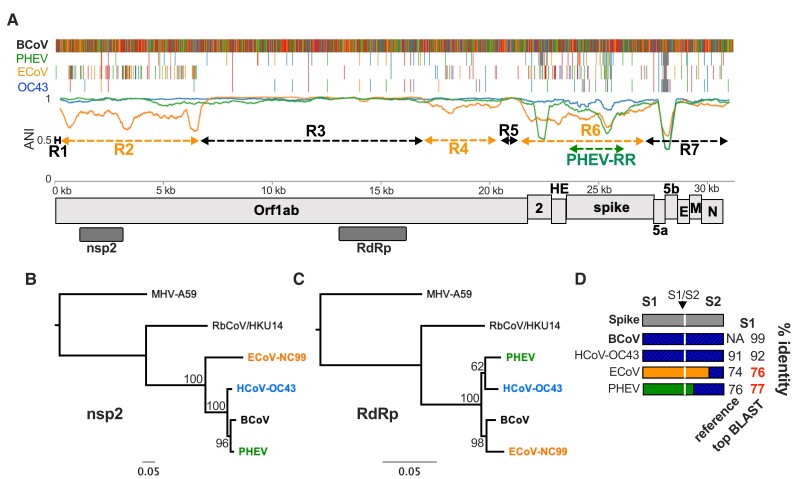
Recombination analysis of *Betacoronavirus-1*. (*A*) Nucleotide identity plot and multiple sequence alignment of *BetaCoV-1* viruses. Orange dashed lines indicate divergent regions of the ECoV-NC99 genome while black dashed lines are regions with high identity to the reference sequence bovine coronavirus (BCoV). (*B*) ML tree of nsp2-encoding region of Orf1ab, which falls within the divergent ECoV-NC99 Region 2. (*C*) ML tree of the RdRp-encoding region of Orf1ab. (*D*) Schematic depicting the spike gene diversity of *BetaCoV1* demonstrating the divergence of ECoV-NC99 and PHEV. Top BLAST hits in bolded red indicate no GenBank entries with >80% nucleotide identity.

We conducted IDPlot analysis of OC43 and these related enzootic viruses of livestock (fig. 4*A*) and identified at least six major recombination breakpoints in the ECoV genome. The largest divergent region (Region 2) is >6 kb (fig. 4*A*). This region encompassing ∼20% of the genome exhibits only ∼75% nt identity to the reference sequence, just ∼81% identity to any known sequence, and occupies a distant phylogenetic position relative to RdRp (fig. 4*B* and *C*, supplementary fig. S6*A, C* and *D*, Supplementary Material online). In contrast to previous reports that ECoV clusters closely with RbCoV/HKU14 in this region (Lau et al. 2012), our analysis suggests that this region of ECoV was acquired via recombination from a viral lineage not documented in GenBank.

Striking variability in ANI within Region 2 led us to conduct a more detailed analysis. IDPlot did not predict internal Region 2 breakpoints, likely because undersampling masks any potential phylogenetic incongruence signal. To determine whether these ANI departures did in fact reflect additional recombination we conducted a manual analysis guided by the IDPlot multiple sequence alignment, phylogenetic trees for each proposed sub-region, and BLAST analysis to further dissect differing evolutionary relationships for sub-regions. We found at least six and possibly seven distinct sub-regions (supplementary fig. S8, Supplementary Material online). Nucleotide identity to top BLAST hits of these sub-regions is highly variable (<70–>90%), as is identity of the hits themselves, with genetic contribution from RbCoV/HKU14-like viruses, BCoV-like viruses, and more distant, uncharacterized lineages within the *Embecovirus* genus (supplementary fig. S5, Supplementary Material online). Due to undersampling, phylogenetic signals were sometimes weakly informative whereas BLAST analysis was more robust. Together, this demonstrates that Region 2 was not acquired via a single recombination event but rather represents a mosaic of known and unknown viral lineages that share an overlapping ecological niche with ECoV.

Another major recombinant ECoV region, Region 6, includes the entire NS2 and conserved HE genes as well as the majority of the spike gene, including most of the slowly evolving, conserved S2 domain (fig. 4*A*, supplementary fig. S6*A*, Supplementary Material online). Within this region on the multiple sequence alignment, we also identified a recombination event encompassing the majority of the PHEV spike gene, though mapping the PHEV breakpoints required downsampling (removing ECoV) to simplify the GARD analysis (fig. 4*A*, supplementary fig. S6*A*, Supplementary Material online). Both ECoV Region 6 and the PHEV recombinant region occupy relatively distant nodes on a phylogenetic tree (supplementary fig. S6*G* and *J*, Supplementary Material online) and exhibit <80% sequence identity to the reference sequence or any sequence in GenBank (supplementary fig. S6*A*, Supplementary Material online). High divergence in the conserved structural genes HE and spike S2 argue strongly in favor of recombination producing this divergence, rather than rapid evolution, as does the uniform dispersion of low identity throughout the region. Additional sampling would produce a more robust phylogenetic signal by further populating the branches containing the ECoV and PHEV spike-encompassing recombinant regions. Finally, we identified a third recombinant region, Region 4, in which ECoV exhibited higher nucleotide identity with RbCoV/HKU14 than with other *BetaCoV1* viruses (supplementary fig. S4*A* and *E*, Supplementary Material online), further demonstrating the highly mosaic nature of the ECoV genome. We conducted an additional analysis to confirm that the IDPlot output is robust to the choice of reference sequence. Substituting OC43 for BCoV as the reference sequence did not alter the IDPlot output (supplementary fig. S7, Supplementary Material online).

Our analyses significantly alter the known evolutionary history of these important viruses. First, previous genomic characterization of ECoV described it as the most divergent member of *BetaCoV1* based on nucleotide identity and phylogenetic positioning of full-length Orf1ab. However, in the >10 kb Region 3 that accounts for ∼1/3 of the entire genome (fig. 4*A*) ECoV exhibits the highest nucleotide identity to BCoV in our dataset (98.5%) (fig. 4*A*, supplementary fig. S6*D*, Supplementary Material online), which is inconsistent with it having diverged earlier than OC43 and PHEV. The latter viruses are estimated to have shared a common ancestor with BCoV 100–150 years ago (Vijgen et al. 2005, 2006), suggesting that all of the observed ECoV recombination has occurred more recently. Second, our discovery of recombinant regions of unknown betacoronavirus origin suggest that unsampled, distantly related lineages occupy overlapping ecological niches with ECoV and may continue to circulate and participate in recombination events. Basal members of the subgenus *Embecovirus*, which includes *BetaCoV1* have been identified exclusively in rodents (fig. 1*B*), suggesting they are a natural reservoir for these viruses. Although relatively little attention has been directed to these viruses outside agricultural settings, studies of BCoV and ECoV cross-neutralization suggest population immunity to OC43 may provide only limited protection against infection mediated by these divergent spikes (Nemoto et al. 2017). No recent zoonotic infections from this lineage have been documented, but the genomic collision of these viruses with yet-undiscovered, presumably rodent viruses warrants a reassessment of their potential threat to human health.

### SADSr-CoVs Encode Highly Diverse Spike and Accessory Genes

**Fig. 5.**
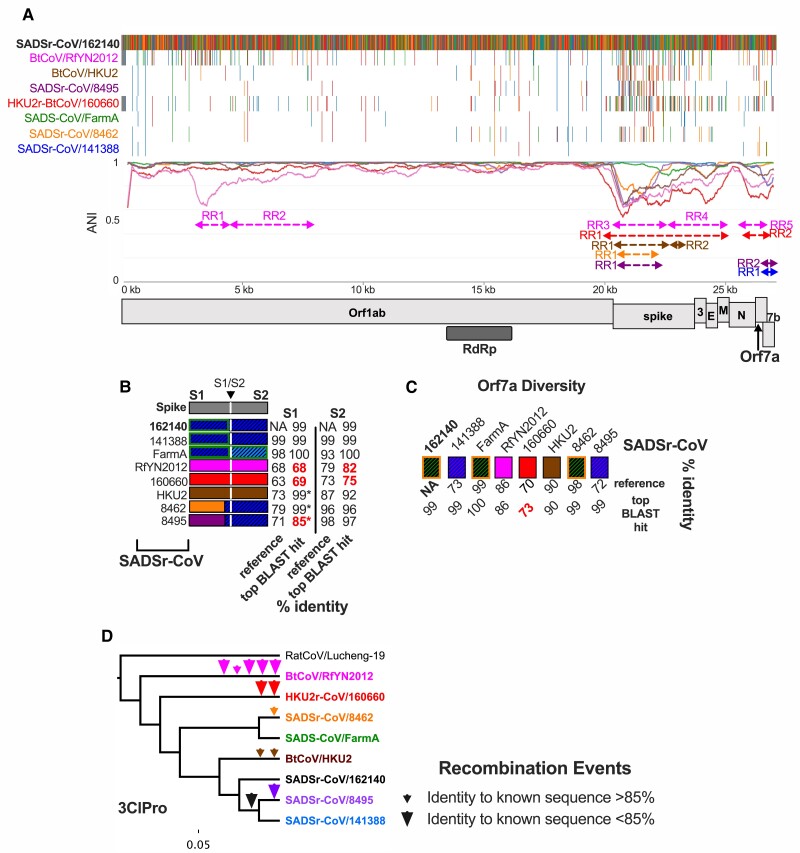
SADSr-CoV IDPlot analysis. (*A*) IDPlot nucleotide identity and multiple sequence alignment of eight SADSr-CoVs. Color-coded dashed lines indicate divergent regions in corresponding viruses owing to recombination events. (*B*) Schematic of spike genes of SADSr-CoVs along with nucleotide identity to the reference sequence and closest related sequences in GenBank for S1 and S2 domains. (*C*) Schematic of Orf7*a* diversity with nucleotide identity to the reference sequence and closest related sequences in GenBank. (*D*) Phylogenetic tree of SADSr-CoVs based on 3ClPro sequence illustrating the history of inferred recombination events indicated by arrowheads.

In 2017 a series of highly lethal diarrheal disease outbreaks on Chinese pig farms were linked to a novel alphacoronavirus, SADS-CoV (Gong et al. 2017; Zhou et al. 2018), which is closely related to the previously described BtCoV/HKU2. Sampling of horseshoe bats nearby affected farms revealed numerous SADSr-CoVs with >95% genome-wide nucleotide identity, suggesting porcine outbreaks were due to spillover from local bat populations. To gain a better view of the genetic diversity among these viruses, we conducted IDPlot analysis of a prototypical SADS-CoV isolate (FarmA) and seven bat SADSr-CoVs sampled at different times before and after the first outbreaks in livestock (fig. 5*A*) using bat SADSr-CoV/162140 as a reference sequence. Three notable observations emerged from the identity plot: 1. Like ECoV, BtCoV/RfYN2012 exhibits evidence of recombination in the 5′ end of Orf1ab, reinforcing that major recombination events are not limited to spike or accessory genes 2. The spike region of the genome (including S2) is highly variable as previously reported (Zhou et al. 2018). The 3′ end of the genome also exhibits considerable diversity (fig. 5*A*).

In the regions of Orf1ab encoding RdRp, 3ClPro, helicase, and methyltransferase NTD-all viruses exhibit 94–100% nucleotide identity to the reference SADSr-CoV/162140 (supplementary figs. S4*E*, *F*, S8*B*–*E*and S9*B*, *C*, Supplementary Material online). In contrast, BtCoV/RfYN2012 RR1 has <70% identity to the reference or any known sequence (supplementary figs. S8*F*and S9*A*, Supplementary Material online), providing evidence that additional uncharacterized alphacoronavirus lineages (of different subgenera) circulates in horseshoe bats, and recombines with SADSr-CoVs.

The spike gene is a striking recombination hotspot among SADSr-CoVs. Due to the clustering of putative breakpoints surrounding the 5′ end, 3′ end, and middle of spike, we ran IDPlot on subsets of three viruses – SADSr-CoV/162140 (reference), SADSr-CoV/141388 or SADS-CoV/FarmA, and a virus of interest from the larger dataset. We found breakpoints delineating six distinct and highly divergent spike genes among the eight analyzed viruses (fig. 5*B*), which reflects recombination events encompassing either the entire spike or the S1 subunit that mediates receptor binding. There are 3 unique full-length spikes (BtCoV/RfY2012, HKU2r-BtCoV/160660, BtCoV/HKU2) with 63–73% nucleotide identity to the reference sequence and two unique S1 domains (SADSr-CoVs/8462 and 8495) with <80% identity to the reference (fig. 5*B*, supplementary fig. S9*A*, Supplementary Material online). Some of these regions match with high identity to partial sequences in GenBank (indicated by an asterisk in fig. 5*B*) which may be either the parent virus of the recombinant spike or different isolates of the same virus for which a full-length genome is available.

In addition to spike, accessory proteins that target innate immunity can play important roles in host range and pathogenesis (Liu et al. 2014). We found a second recombination hotspot surrounding the accessory gene Orf7a, which rivals spike gene diversification. Specifically, our dataset contained five distinct Orf7a genes, some of which lack any closely related sequences in GenBank (fig. 5*C*, supplementary fig. S9*A*, Supplementary Material online).

Finally, we mapped each inferred occurrence of a recombination event onto a SADSr-CoV phylogenetic tree. SADSr-CoVs 141388 and 8495 share an Orf7a recombination event, suggesting a recent common ancestor for these two viruses. The tree based on 3ClPro was most consistent with this evolutionary scenario (fig. 5*D*), while the other trees exhibit slightly different topology with minimal diversity, likely due to cryptic recombination events among very closely related viruses. Considering the 3ClPro tree, it is evident that many independent recombination events occurred in the very recent past given that few of the events are shared among the viruses in our dataset (fig. 5*D*).

The SADSr-CoV lineage is rapidly diversifying via recombination, particularly in the spike and ORF7a accessory genes. We observed that numerous viruses with >95–99% identity in conserved Orf1ab regions contain highly divergent spike and accessory genes which may shift host range and virulence in otherwise nearly isogenic viruses. These findings highlight how viruses sampled to date represent only a sliver of circulating SADSr-CoV coronavirus diversity and that coronaviruses can change rapidly, drastically, and unpredictably via recombination with both known and unknown lineages. The SADSr-CoVs exemplify the potential of coronaviruses to rapidly evolve through promiscuous recombination.

To test if deviations in ANI reflected true recombination events producing robust breakpoint predictions, we conducted simulations in which randomly generated variation was introduced into the reference sequence (SADSr-CoV/162140) at rates of 3, 6, or 9% and then ran GARD with query sequences SADS-CoV/FarmA and SADSr-CoV/8495 (supplementary fig. S10*A*–*C*, Supplementary Material online). Random variation erased signals of localized ANI divergence observed with the genuine sequences. With the simulated sequences, GARD ran a maximum of five iterations and called breakpoints even without drops in ANI. Breakpoints were evenly distributed across the genome with no enrichment in regions encompassing the spike or accessory genes (supplementary fig. S10*D*, Supplementary Material online). This analysis supports our approach validating GARD predictions with ANI deviations to greatly enrich for calling true recombination events. Simulations also support our new approach implemented in IDPlot of generating AIC curves to filter out noise of spurious breakpoints from GARD analysis by selecting iterations that do not overfit the sequence comparisons.

## Discussion

We developed IDPlot to explore the role of recombination in the diversification of coronaviruses. Coronaviruses are ubiquitous human pathogens with vast and underexplored genetic diversity. SARS-CoV-2 is the second SARSr-CoV known to infect humans and the fifth zoonotic coronavirus known to sweep through the human population following HCoVs 229E, NL63, HKU1, and OC43. Most effort in evaluating the threat to human health posed by coronaviruses has been dedicated to discovery of novel SARSr-CoVs in wildlife, yet prior to the SARS-CoV-2 pandemic this group of viruses went largely undetected. Much less attention has been paid to other groups that have produced human coronaviruses such as the sparsely sampled *Betacoronavirus-1* and emerging livestock viruses such as the SADSr-CoVs, which exhibit potential to infect humans and already have significant economic impacts.

Recombination detection can be difficult when parental viruses are unknown, as was revealed with our analysis, due to difficulty in distinguishing between true recombination events versus repeated mutations under strong selective pressure. Rapid evolution is most evident for spike receptor-binding domains, leading to polymorphism at critical residues (Li 2016; Guo et al. 2020). Multiple sequence alignments generated by IDPlot demonstrate that even in divergent S1 domains, the low nucleotide identity is evenly distributed throughout putative recombinant regions. Many of the recombination events encompassing S1 included flanking regions of conserved upstream and downstream genes, bolstering the likelihood these represent true recombination events. In all of these regions, including accessory genes, reshuffling of phylogenetic trees described in our analysis provides strong evidence that recombination, not repeated individual mutations of critical amino acid residues, accounts for the observed diversity.

We initially used the SARS-CoV-2-like viruses to test and validate IDPlot and in the process characterized recombination among these viruses in greater detail than previously reported. The observed variability in arrangements of PangolinCoV/GD19 and RmYN02 on a *SARSr-CoV* phylogenetic tree (fig. 3*B*, *C* and *E*, supplementary fig. S5, Supplementary Material online) depending on the region being sampled is a classic recombination signal easily observed in the IDPlot output. We also analyzed recombination dynamics for viruses in *BetaCoV1* and among SADSr-CoVs. Broad similarities emerge from these studies. Most recombination appears to involve the spike gene and/or various accessory genes. However, in both *BetaCoV1* and among SADSr-CoVs we detected recombination events in Orf1ab as well. Spike and accessory gene recombination events are particularly notable given the potential to influence host range and pathogenesis.

This preliminary analysis showed that IDPlot is a powerful new pipeline for sequence identity analysis, breakpoint prediction, and phylogenetic analysis. Existing workflows for nucleotide similarity analysis are proprietary, lack the ability to identify phylogenetic incongruence that is a signature of recombination and do not support direct export of genomic regions for BLAST analysis. This automates and streamlines multi-step analysis with few barriers to use. Nevertheless, there are opportunities for further improvement. We downsampled repeatedly because of the difficulty of resolving breakpoints in several sequences clustered in close proximity in the multiple sequence alignment, as observed surrounding and within SADSr-CoV and other spike genes. Second, GARD is computationally intensive and best suited to small data sets. It is configured as an optional step in IDPlot, so multiple sequence alignments and nucleotide identity plots can be rapidly generated in a local environment. However, for GARD analysis we relied on a high-performance computing cluster to expedite the process. In the future, we anticipate adding other, less intensive breakpoint prediction algorithms to the IDPlot options menu. Future advances in computational methods may also improve the ability to resolve unique breakpoints clustered in genomic regions that are recombination hotspots, most notably the spike gene.

Our IDPlot analyses revealed new evidence of extensive recombination-driven evolution in other coronavirus groups. Wildlife sampling indicates that SADSr-Covs are a large pool of closely related viruses circulating in horseshoe bat populations at high frequency. This is the same genus of bats that include SARSr-CoVs suggesting that the ecological conditions for SADSr-CoV spillover into humans may be in place. The relatedness of these viruses means they have had little time to diverge via mutation, but we find they are rapidly diversifying due to recombination, acquiring spike and accessory genes from unsampled viral lineages. These findings demonstrate that rather than a single threat to human health posed by SADS-CoV, there is a highly diverse reservoir of such viruses in an ecological position and with diversity reminiscent of SARSr-CoVs. We found a similar dynamic at play among *BetaCoV1* which are undersampled to an even greater degree and receive far less attention. Nevertheless, these viruses are involved in genetic exchange with unsampled lineages, with unpredictable consequences.

Our findings bear on strategies for anticipating and countering future zoonotic events. SARSr-CoVs garner considerable attention, with an intense focus on viruses able to infect human cells using ACE-2 as an entry receptor. However, RmYN02 demonstrates that viruses can toggle between spikes that recognize ACE-2 or different entry receptors but still infect the same hosts and continue to undergo recombination. Work to prepare for future zoonotic SARSr-CoVs must account for the possibility that the threat will come from coronaviruses only distantly related to SARSr-CoVs undergoing frequent recombination and distributing genetic diversity across the phylogenetic tree of coronaviruses.

More attention to the evolutionary dynamics of *BetaCoV1* and SADSr-CoVs is also warranted. Both groups originate in wildlife: rodents and horseshoe bats, respectively, and are enzootic or epizootic in livestock. *BetaCoV1* includes a pandemic virus that swept the human population, OC43, while SADS-CoV efficiently infects primary human respiratory and intestinal epithelial cells (Edwards et al. 2020). Increased surveillance at wildlife-livestock interfaces, including agricultural workers is needed for early detection of novel viruses coming into contact with humans. Due to recombination, prior infection with a virus such as OC43 cannot be presumed to be protective against even closely related viruses that can encode highly divergent spikes, as demonstrated in our analysis. Similarly, efforts to develop medical countermeasures against SADS-CoV should consider the full breadth of diversity among related viruses, while aiming for broadly effective vaccines and therapeutics.

Using IDPlot, we identified extensive diversity among coronavirus spike and accessory genes with potential implications for future pandemics. From the standpoint of understanding coronavirus evolution, frequent recombination events often reshuffle phylogenetic trees and can obscure evolutionary relationships. The extent to which viruses in current databases contain genomic regions with no known close relatives makes clear that coronavirus diversity is vast and poorly sampled, even for viruses circulating in well-studied locations. This proximity raises the possibility of recurrent zoonoses of coronaviruses encoding divergent spike and accessory genes. Therefore, preparedness efforts should consider a broad range of virus diversity rather than risk a more narrow focus on close relatives of coronaviruses that most recently impacted human health.

## Methods

### Virus Sequences

All sequences were downloaded from GenBank with the exception of PangolinCoV/GD19 and BtCoV/RmYN02, which were acquired from the Global Initiative on Sharing All Influenza Data (GISAID) database (https://www.gisaid.org) and analyzed with the permission of Edward C. Holmes on behalf of all authors.

### IDPlot

The development of IDPlot, a series of steps implemented in Nextflow, was motivated by reviewing the pain points of installing and running a common series of tools for sequence comparison. We aimed to create something very simple to use that required as little user experience as possible while still supporting a complex analysis. In doing so, we created a Nextflow pipeline that requires just two FASTA files—one FASTA file containing a sequence the user has identified as the reference sequence (the sequence that will be most prominent in the report) and another FASTA file containing one or many additional sequences to compare to the “reference” sequence. With Nextflow and Docker installed on the user's system, the IDPlot workflow can be run on these two FASTA files using the following:

nextflow run brwnj/idplot -latest -with-docker \

--reference references/MN996532.fasta \
--fasta ‘queries/*.fasta’

To include breakpoint detection via GARD this becomes:

nextflow run brwnj/idplot -latest -with-docker

--reference references/MN996532.fasta
--fasta ‘queries/*.fasta’
--**gard**

A GFF3 annotation file may also be provided to display reference genome annotations such as gene intervals.

nextflow run brwnj/idplot -latest -with-docker \

--reference references/MN996532.fasta \
--fasta ‘queries/*.fasta’ \
--gard \

**--gff MN996532.gff3**

Since IDPlot utilizes Nextflow, the workflow and dependency deployment are handled entirely in the background. The analysis steps of read alignment via MAFFT, breakpoint detection with GARD, tree generation using FastTree2, and report compilation in Python run without further user intervention on local, high-performance compute, or cloud systems.

The final output of IDPlot includes the output from all intermediate steps organized by algorithm in the results directory. The powerful aspect of the workflow resides in its interactive HTML report, which facilitates analysis of GARD's iterative model fit improvements, phylogenetic tree viewing per breakpoint (per GARD iteration), and a plot of ANI. ANI is calculated across a sliding window (default: 500 bp) with the value being plotted at the window's center point. The report further allows the user to quickly snip a section of DNA sequence and send it to NCBI's BLAST to identify sequence relatives. More details on the usage of IDPlot and its output are provided in the code repository at https://github.com/brwnj/idplot.

### Phylogenetic Validation of Breakpoints

Putative breakpoints were further tested by maximum-likelihood phylogenetic analysis using PhyML (Guindon et al. 2005). For *Betacoronavirus-1*, RbCoV/HKU14 and MHV (as a root) were aligned with the four viruses in the IDPlot dataset. For SADSr-CoVs we chose HCoV-229E as the root, with the exception of the spike gene, and aligned it with the eight viruses in our dataset. We rooted the SARSr-CoVs with BtCoV/BM48–31/BGR/2008. Given the better sampling of SARSr-CoV, we included more diversity in that alignment to enhance phylogenetic signal. The signal for *BetaCoV1* and SADSr-CoV is constrained by sampling limitations. We extracted breakpoint-defined regions from the alignment and generated ML phylogenetic trees using a GTR substitution model and 100 bootstraps. “Up” and “Dn” regions are the 500 nucleotides upstream or downstream of a proposed 5′ or 3′ breakpoint, respectively. In the case of SADSr-CoV the clustering of breakpoints around the 5′ and 3′ ends of spike precluded using unique Up and Dn regions for each recombination event. Instead, we used the N-terminal section of nsp16 (MTase) and the M gene, respectively. For BtCoV/RmYN02 RR2 and ORf8 phylogenetic testing we excluded SARSr-CoVs that have a deletion in Orf8. The RmYN02 UpRR2 alignment also does not include BtCoV/WIV1 because it has a unique open reading frame inserted in this region and so does not align with SARSr-CoVs lacking this Orfx.

### BLAST Analysis

To identify the source of recombinant regions identified by GARD, we used NCBI Blastn with default parameters, excluding the query sequence from the search. For SADSr-CoVs partial spike sequences frequently appear as top hits. We included these, denoted by an asterisk in reporting the results.

### Graphical Depictions of Spike Recombination Events

Figures 3 *D* and *D*, and 5*B* are graphical depictions of the spike recombination events inferred by GARD and delineated on the ANI plot and in associated phylogenetic trees, both in the main as well as supplementary figures. They were manually constructed using drawing and coloring functions in GraphPad Prism. The blue box with black hatching represents the spike gene of the reference sequence. Colors of spikes with recombined regions match the color of the respective virus in the IDPlot output, and size of recombined regions roughly correlate with the location of the recombination event within or incorporating the indicated spike.

### Simulations

Multiple sequence alignment of MF094688, MF094687, MF094686 reference sequences using MAFFT resulted in an alignment of length 27,200 bp with MF094687 and MF094686 differing by 305 bp (1.12%) and 967 bp (3.56%), including gaps, respectively. Errors were introduced randomly using NumPy (random.uniform) into our reference sequence MF094688 (27,177 bp) at rates of 3% (815 bp), 6% (1,631 bp), and 9% (2,446 bp) to simulate non-biologically driven mutation. Bases were changed to a random base among the remaining three. No effort was made with respect to reading frame or other biological factors with respect to base changes. 1,000 pairs of sequences at each error rate were aligned against MF094688 and breakpoints were identified using GARD.

## Supplementary Material

evac161_Supplementary_Data

## Data Availability

The raw data underlying these analyses are available on FigShare at the following link: https://figshare.com/articles/dataset/Untitled_Item/21350175.
