# Supplementary material for: Extensive Recombination-driven Coronavirus Diversification Expands the Pool of Potential Pandemic Pathogens
Source: Genome Biol Evol. 2022 Nov 4;14(12):evac161. doi: 10.1093/gbe/evac161 (PMC9730504; doi:10.1093/gbe/evac161)
Supplement: evac161_Supplementary_Data [file evac161_supplementary_data.pdf]

Accession Number	Virus	Accession Number	Virus
MK581202	IBV/Poland/80/1989	MN996532	BtCoV/RaTG13
KF294380	RnCoV/Lucheng-19	MN908947	SARS-CoV-2
MF577027	PEDV/Belgorod/2008	KJ473811	BtCoV/RfJL2012
AY567487	HCoV-NL63	MG772934	BtCoV/ZXC21
MN611517	229Er-CoV/Ra5425	MG772933	BtCoV/ZC45
AF304460	HCoV-229E	KY417145	BtCoV/Rf4092
KJ473808	BtCoV/RfYN2012	KF367457	BtCoV/WIV1
MN611512	HKU2r-BtCoV/160660	AY278741	SARS-CoV/Urbani
NC_009988	BtCoV/HKU2	MT350598	HKU9r-COV/GCCDC1
MF094685	SADSr-CoV/8462	MG762674	BtCoV/HKU9
MF094687	SADSr-CoV/141388	AY700211	MHV-A59
MF094681	SADS-CoV/FarmA	KF686346	HCoV-HKU1
MF094688	SADSr-CoV/162140	NC_046954	RfMfCoV-2/JL2014
MF094686	SADSr-CoV/8495	MH687976	VZ-BetaCoV/22084-1
KC869678	NeoCoV/PML-PHE-1/RSA2011	NC_026011	RnCoV/HKU24
JX869059	MERS_CoV	KY370052	RtMmCoV-1/IM2014
EF065505	BtCoV/HKU4	KY370044	RtAsCoV/IM2014
MH002342	BtCoV/HKU5	JN874559	RbCoV/HKU14
KY352407	BtCoV/KY72	DQ011855	PHEV
NC_014470	BtCoV/BM48-31/BGR/2008	AY391777	HCoV-OC43
EPI_ISL_410538	PangolinCoV/GX17-P4L	EF446615	ECoV-NC99
EPI_ISL_410721	PangolinCoV/GD19	MH043952	BCoV
EPI_ISL_412977	BtCoV/RmYN02		

Figure S1. Accession numbers of viral sequences analyzed

A

	BCoV	HCoV-OC43	PHEV	ECoV-NC99	RbCoV/HK...
BCoV		97.5%	97.3%	97.8%	90.9%
HCoV-OC43	97.5%		97.7%	96.8%	90.5%
PHEV	97.3%	97.7%		96.6%	90.7%
ECoV-NC99	97.8%	96.8%	96.6%		90.3%
RbCoV/HKU14	90.9%	90.5%	90.7%	90.3%	

B

	162140	141388	HKU2	FarmA	8495	8462	160660	RfYN2012
162140		99.3%	97.6%	99.5%	99.3%	97.6%	95.3%	94.4%
141388	99.3%		97.8%	99.2%	98.8%	98.0%	95.3%	94.5%
HKU2	97.6%	97.8%		97.6%	97.4%	98.4%	95.1%	94.8%
FarmA	99.5%	99.2%	97.6%		99.1%	97.7%	95.1%	94.4%
8495	99.3%	98.8%	97.4%	99.1%		97.4%	95.2%	94.3%
8462	97.6%	98.0%	98.4%	97.7%	97.4%		94.9%	94.2%
160660	95.3%	95.3%	95.1%	95.1%	95.2%	94.9%		94.6%
RfYN2012	94.4%	94.5%	94.8%	94.4%	94.3%	94.2%	94.6%	

C

	SARS-CoV-2	RmYN02	RaTG13	PangolinC...
SARS-CoV-2		98.4%	97.8%	91.3%
RmYN02	98.4%		97.4%	91.4%
RaTG13	97.8%	97.4%		90.7%
PangolinCoV/GD19	91.3%	91.4%	90.7%	

Figure S2. Distance tables of full-length RdRp coding region of Orf1ab. A) Distance table for *Betacoronavirus-1* RdRp sequences, as well as RbCoV/HKU14. B) Distance table for SADSr-CoV RdRp.C) Distance table for RdRp coding region of SARS-CoV-2-like viruses.

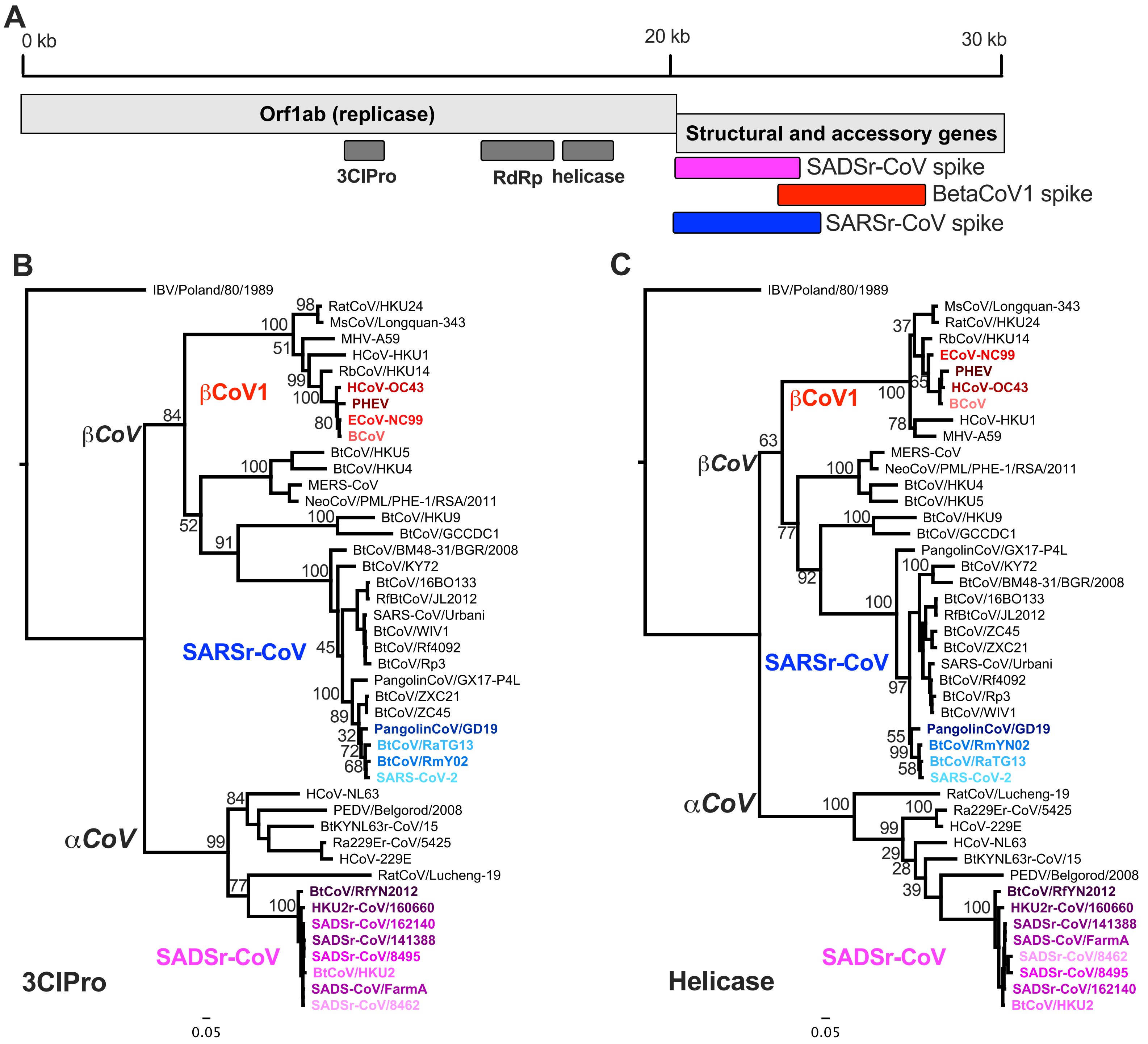

Figure S3. 3CIPro and Helicase phylogenetic trees. A) Schematic showing position of Orf1ab regions encoding 3CIPro, RdRp, and Helicase B) Maximum-likelihood phylogenetic tree of 3CIPro-encoding region. C) ML tree of helicase-encoding region of Orf1ab.

A

3CLPro

	BCoV	HCoV-OC43	PHEV	ECoV-NC99	RbCoV/HK...
BCoV		96.6%	95.3%	99.2%	87.9%
HCoV-OC43	96.6%		94.7%	96.7%	87.8%
PHEV	95.3%	94.7%		95.2%	86.7%
ECoV-NC99	99.2%	96.7%	95.2%		88.1%
RbCoV/HKU14	87.9%	87.8%	86.7%	88.1%	

B

Helicase

	BCoV	HCoV-OC43	PHEV	ECoV-NC99	RbCoV/HK...
BCoV		97.4%	95.6%	92.3%	88.6%
HCoV-OC43	97.4%		96.0%	91.5%	88.5%
PHEV	95.6%	96.0%		90.3%	87.7%
ECoV-NC99	92.3%	91.5%	90.3%		92.5%
RbCoV/HKU14	88.6%	88.5%	87.7%	92.5%	

C

3CLPro

	SARS-CoV-2	RmYN02	RaTG13	PangolinC...
SARS-CoV-2		96.9%	95.9%	93.8%
RmYN02	96.9%		95.8%	93.1%
RaTG13	95.9%	95.8%		92.9%
PangolinCoV/GD19	93.8%	93.1%	92.9%	

D

Helicase

	SARS-CoV-2	RmYN02	RaTG13	PangolinC...
SARS-CoV-2		97.5%	98.1%	92.2%
RmYN02	97.5%		97.8%	92.1%
RaTG13	98.1%	97.8%		92.2%
PangolinCoV/GD19	92.2%	92.1%	92.2%	

E

3CLPro

	162140	141388	HKU2	FarmA	8495	8462	BtCoV/16...	RfYN2012
162140		99.8%	98.8%	99.3%	99.9%	99.3%	97.0%	95.6%
141388	99.8%		98.7%	99.1%	99.9%	99.1%	97.0%	95.4%
HKU2	98.8%	98.7%		98.6%	98.8%	98.6%	96.6%	95.3%
FarmA	99.3%	99.1%	98.6%		99.2%	99.8%	97.2%	95.5%
8495	99.9%	99.9%	98.8%	99.2%		99.2%	97.1%	95.5%
8462	99.3%	99.1%	98.6%	99.8%	99.2%		97.2%	95.5%
BtCoV/160660	97.0%	97.0%	96.6%	97.2%	97.1%	97.2%		95.3%
RfYN2012	95.6%	95.4%	95.3%	95.5%	95.5%	95.5%	95.3%	

F

Helicase

	162140	141388	FarmA	HKU2	8495	8462	BtCoV/16...	RfYN2012
162140		99.0%	99.1%	98.7%	95.6%	96.2%	95.1%	93.9%
141388	99.0%		99.8%	98.4%	96.3%	96.3%	95.1%	93.5%
FarmA	99.1%	99.8%		98.4%	96.2%	96.4%	95.1%	93.4%
HKU2	98.7%	98.4%	98.4%		95.9%	96.3%	95.6%	94.3%
8495	95.6%	96.3%	96.2%	95.9%		96.4%	94.1%	93.1%
8462	96.2%	96.3%	96.4%	96.3%	96.4%		94.1%	93.1%
BtCoV/160660	95.1%	95.1%	95.1%	95.6%	94.1%	94.1%		94.5%
RfYN2012	93.9%	93.5%	93.4%	94.3%	93.1%	93.1%	94.5%	

Figure S4. 3CLPro and Helicase genetic distance tables. A and B) Distance tables for BetaCoV1 viruses. C and D) Distance tables for SARS-CoV-2-like viruses. E and F) Distance tables for SADSr-CoVs.

A

Region	Position (in native sequence)	% identity to reference	Top BLAST hit (% identity)
PangolinCoV/GD19 RR1	21,431-22,321	64.2%	MG772934.1 – BtCoV/ZXC21 (80.4%)
BtCoV/RmYN02 RR1	21,424-24,102	62.5%	KY417145.1 – BtCoV/Rf4092 (73.8%)
BtCoV/RmYN02 RR2	27,700-28,078	51.9%	AY515512.1 – civetSARS-CoV (81%)

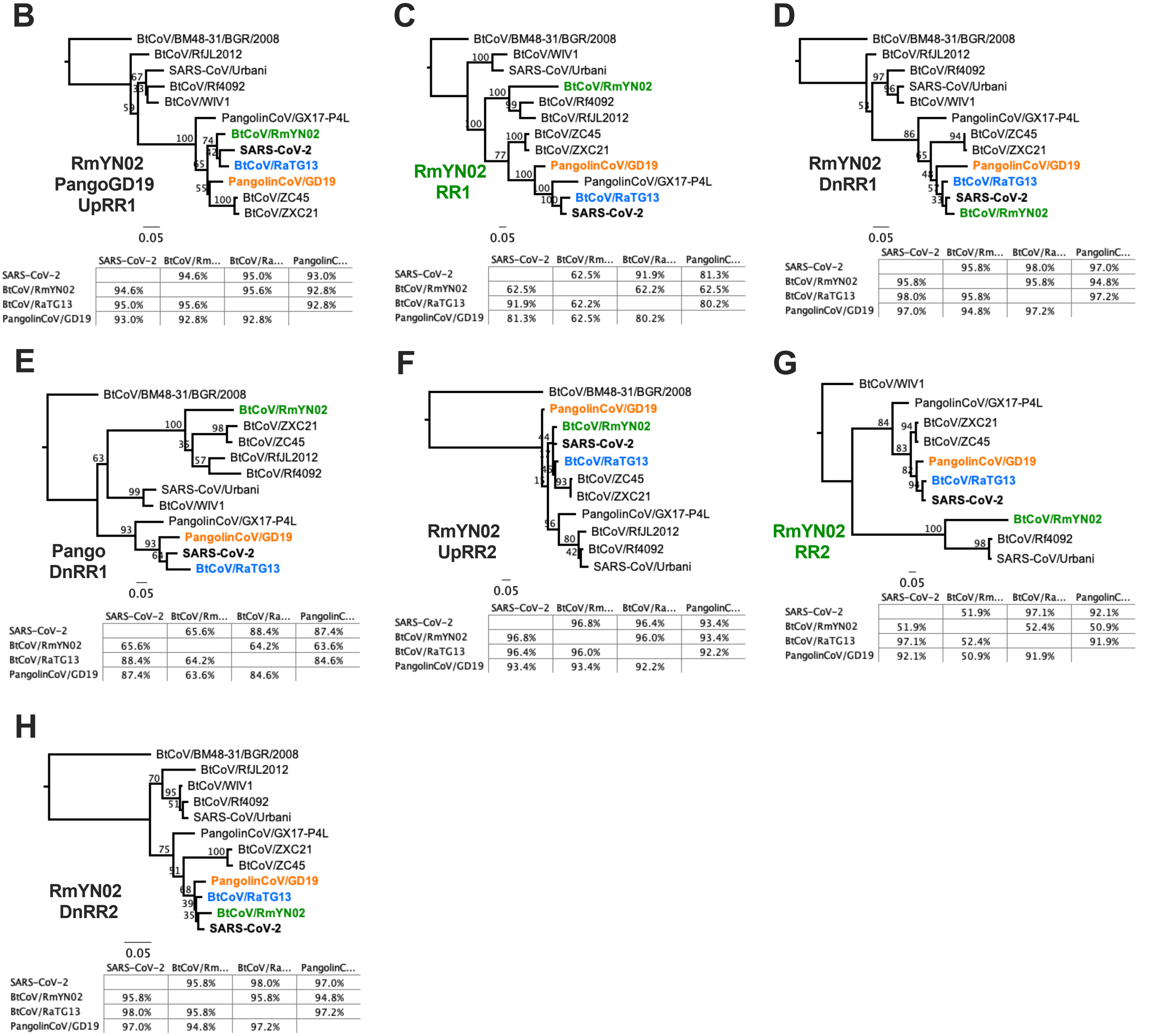

B (RmYN02 PangoGD19 UpRR1)

	SARS-CoV-2	BtCoV/Rm...	BtCoV/Ra...	PangolinC...
SARS-CoV-2		94.6%	95.0%	93.0%
BtCoV/RmYN02	94.6%		95.6%	92.8%
BtCoV/RaTG13	95.0%	95.6%		92.8%
PangolinCoV/GD19	93.0%	92.8%	92.8%	

C (RmYN02 RR1)

	SARS-CoV-2	BtCoV/Rm...	BtCoV/Ra...	PangolinC...
SARS-CoV-2		62.5%	91.9%	81.3%
BtCoV/RmYN02	62.5%		62.2%	62.5%
BtCoV/RaTG13	91.9%	62.2%		80.2%
PangolinCoV/GD19	81.3%	62.5%	80.2%	

D (RmYN02 DnRR1)

	SARS-CoV-2	BtCoV/Rm...	BtCoV/Ra...	PangolinC...
SARS-CoV-2		95.8%	98.0%	97.0%
BtCoV/RmYN02	95.8%		95.8%	94.8%
BtCoV/RaTG13	98.0%	95.8%		97.2%
PangolinCoV/GD19	97.0%	94.8%	97.2%	

E (Pango DnRR1)

	SARS-CoV-2	BtCoV/Rm...	BtCoV/Ra...	PangolinC...
SARS-CoV-2		65.6%	88.4%	87.4%
BtCoV/RmYN02	65.6%		64.2%	63.6%
BtCoV/RaTG13	88.4%	64.2%		84.6%
PangolinCoV/GD19	87.4%	63.6%	84.6%	

F (RmYN02 UpRR2)

	SARS-CoV-2	BtCoV/Rm...	BtCoV/Ra...	PangolinC...
SARS-CoV-2		96.8%	96.4%	93.4%
BtCoV/RmYN02	96.8%		96.0%	93.4%
BtCoV/RaTG13	96.4%	96.0%		92.2%
PangolinCoV/GD19	93.4%	93.4%	92.2%	

G (RmYN02 RR2)

	SARS-CoV-2	BtCoV/Rm...	BtCoV/Ra...	PangolinC...
SARS-CoV-2		51.9%	97.1%	92.1%
BtCoV/RmYN02	51.9%		52.4%	50.9%
BtCoV/RaTG13	97.1%	52.4%		91.9%
PangolinCoV/GD19	92.1%	50.9%	91.9%	

H (RmYN02 DnRR2)

	SARS-CoV-2	BtCoV/Rm...	BtCoV/Ra...	PangolinC...
SARS-CoV-2		95.8%	98.0%	97.0%
BtCoV/RmYN02	95.8%		95.8%	94.8%
BtCoV/RaTG13	98.0%	95.8%		97.2%
PangolinCoV/GD19	97.0%	94.8%	97.2%	

Figure S5. Breakpoint and phylogenetic analysis of *SARSr-CoV*. A. Table showing boundaries of divergent recombinant regions in PangolinCoV/GD19 and BtCoV/RmYN02 with nucleotide identity to SARS-CoV-2 and the top BLAST hit in GenBank. B-H) Maximum-likelihood phylogenetic trees of breakpoint-defined regions in PangolinCoV/GD19 and BtCoV/RmYN02, demonstrating changes in topology due to recombination. "Up" and "Dn" regions reflect the 500 nt upstream and downstream of 5' and 3' breakpoints, respectively.

A

Region	Position (in native sequence)	% identity to reference	Top BLAST hit (% identity)
ECoV R2	330-6,752	75.4	JN874560.1 - RbCoV/HKU14 (80.9)
ECoV R4	17,025-20,320	87.8	JN874561.1 – RbCoV/HKU14 (91.3)
ECoV R6	21,496-27,145	75.8	LC494155.1 - BCoV (76.5)
PHEV RR	23,219-25,931	77.1	LC494159.1 - BCoV (77.8)

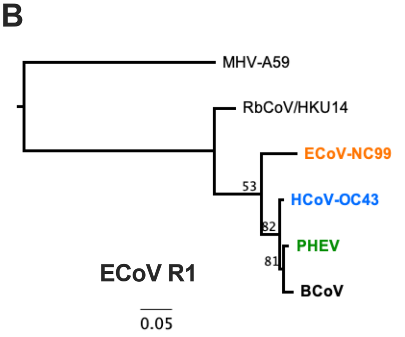

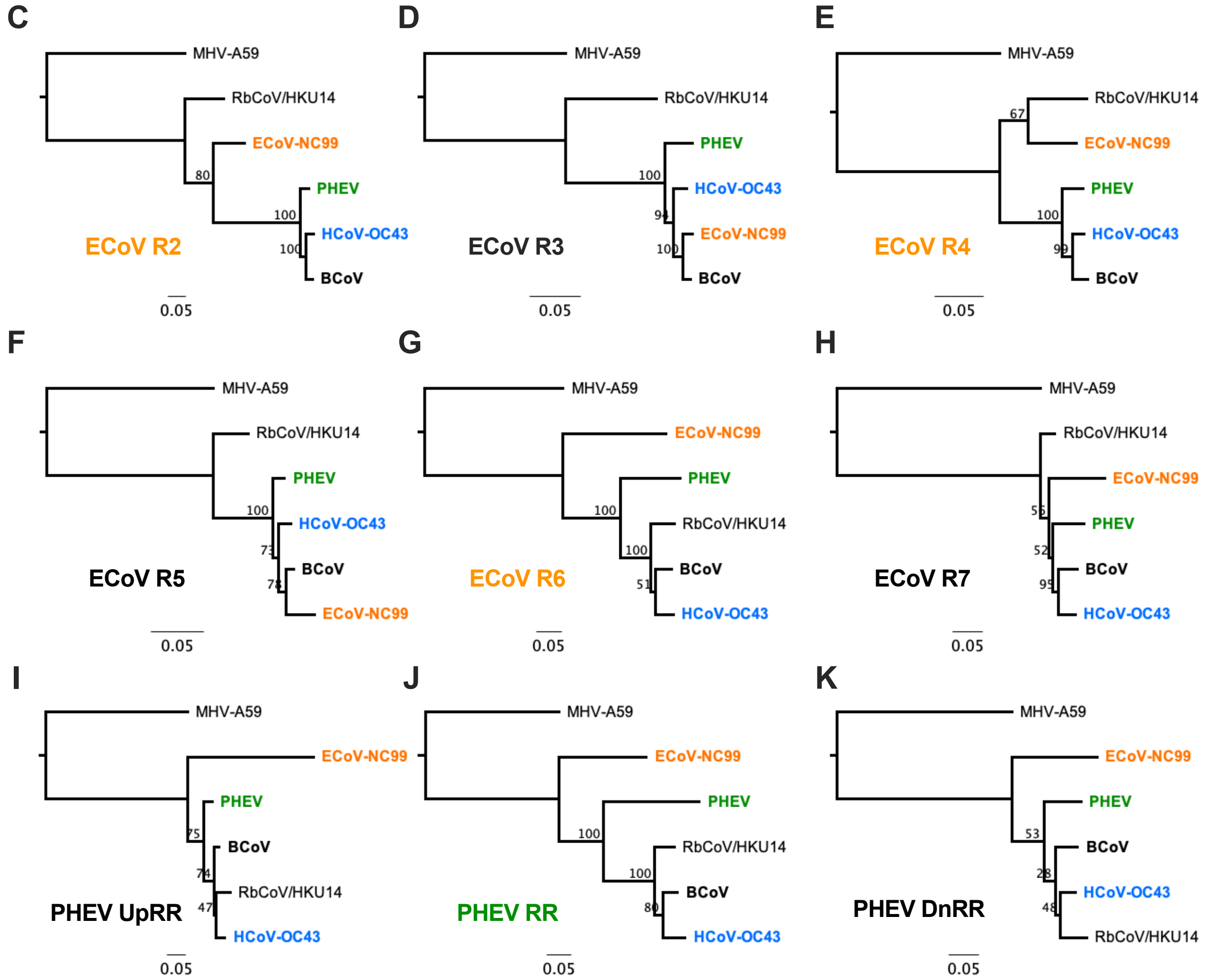

Figure S6. Breakpoint and phylogenetic analysis of *Betacoronavirus-1*. A. Table showing boundaries of divergent recombinant regions in ECoV and PHEV with nucleotide identity to BCoV and the top BLAST hit in GenBank. B-H) Maximum-likelihood phylogenetic trees of breakpoint-defined regions in ECoV. I-K) ML-phylogenetic trees of PHEV-RR and 500 nucleotide stretches flanking the 5' and 3' breakpoints. ECoV and PHEV regions in black type are conserved among *BetaCoV1* (not of recombinant origin) and demonstrate changes in tree topology and/or branch length due to recombination.

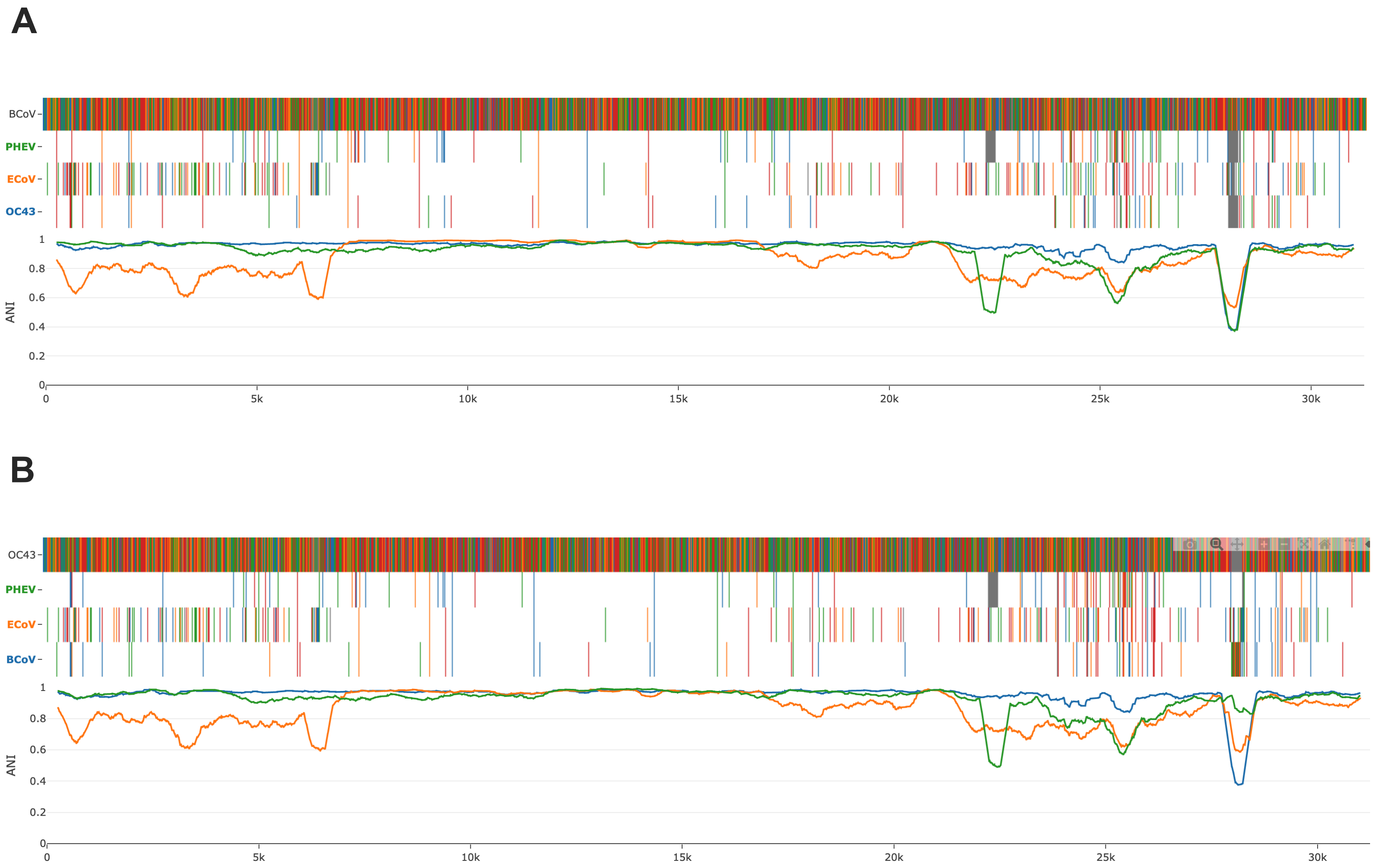

Figure S7. BetaCoV-1 IDPlot results with alternative reference sequences. A) The IDPlot output for *Betacoronavirus-1* displayed in Figure 4 of the main text. B) The IDPlot output from a run using the same sequences but with OC43 as the reference rather than BCoV.

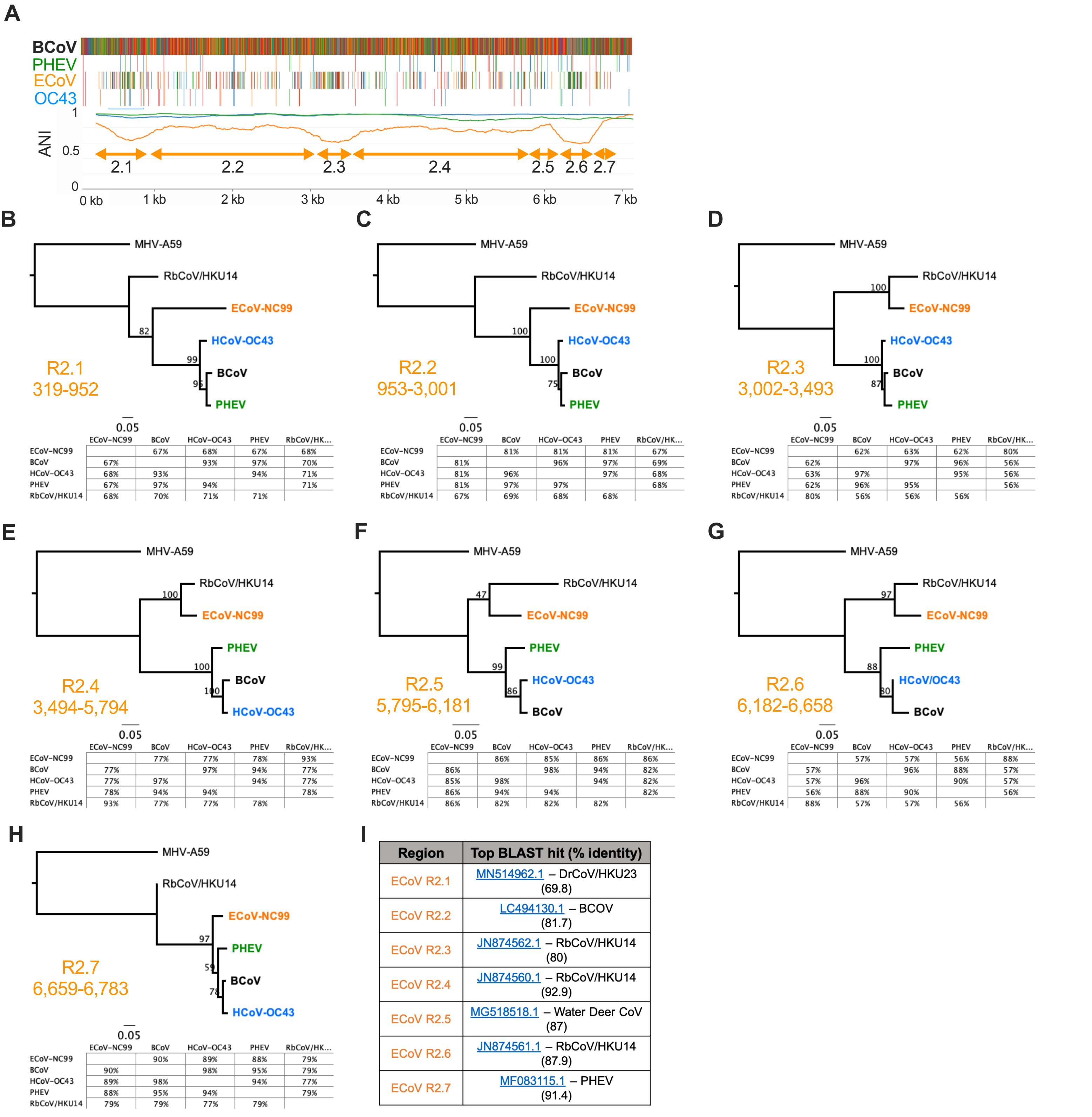

B (R2.1, 319-952)

	ECoV-NC99	BCoV	HCoV-OC43	PHEV	RbCoV/HK...
ECoV-NC99		67%	68%	67%	68%
BCoV	67%		93%	97%	70%
HCoV-OC43	68%	93%		94%	71%
PHEV	67%	97%	94%		71%
RbCoV/HKU14	68%	70%	71%	71%	

C (R2.2, 953-3,001)

	ECoV-NC99	BCoV	HCoV-OC43	PHEV	RbCoV/HK...
ECoV-NC99		81%	81%	81%	67%
BCoV	81%		96%	97%	69%
HCoV-OC43	81%	96%		97%	68%
PHEV	81%	97%	97%		68%
RbCoV/HKU14	67%	69%	68%	68%	

D (R2.3, 3,002-3,493)

	ECoV-NC99	BCoV	HCoV-OC43	PHEV	RbCoV/HK...
ECoV-NC99		62%	63%	62%	80%
BCoV	62%		97%	96%	56%
HCoV-OC43	63%	97%		95%	56%
PHEV	62%	96%	95%		56%
RbCoV/HKU14	80%	56%	56%	56%	

E (R2.4, 3,494-5,794)

	ECoV-NC99	BCoV	HCoV-OC43	PHEV	RbCoV/HK...
ECoV-NC99		77%	77%	78%	93%
BCoV	77%		97%	94%	77%
HCoV-OC43	77%	97%		94%	77%
PHEV	78%	94%	94%		78%
RbCoV/HKU14	93%	77%	77%	78%	

F (R2.5, 5,795-6,181)

	ECoV-NC99	BCoV	HCoV-OC43	PHEV	RbCoV/HK...
ECoV-NC99		86%	85%	86%	86%
BCoV	86%		98%	94%	82%
HCoV-OC43	85%	98%		94%	82%
PHEV	86%	94%	94%		82%
RbCoV/HKU14	86%	82%	82%	82%	

G (R2.6, 6,182-6,658)

	ECoV-NC99	BCoV	HCoV-OC43	PHEV	RbCoV/HK...
ECoV-NC99		57%	57%	56%	88%
BCoV	57%		96%	88%	57%
HCoV-OC43	57%	96%		90%	57%
PHEV	56%	88%	90%		56%
RbCoV/HKU14	88%	57%	57%	56%	

H (R2.7, 6,659-6,783)

	ECoV-NC99	BCoV	HCoV-OC43	PHEV	RbCoV/HK...
ECoV-NC99		90%	89%	88%	79%
BCoV	90%		98%	95%	79%
HCoV-OC43	89%	98%		94%	77%
PHEV	88%	95%	94%		79%
RbCoV/HKU14	79%	79%	77%	79%	

I

Region	Top BLAST hit (% identity)
ECoV R2.1	MN514962.1 – DrCoV/HKU23 (69.8)
ECoV R2.2	LC494130.1 – BCOV (81.7)
ECoV R2.3	JN874562.1 – RbCoV/HKU14 (80)
ECoV R2.4	JN874560.1 – RbCoV/HKU14 (92.9)
ECoV R2.5	MG518518.1 – Water Deer CoV (87)
ECoV R2.6	JN874561.1 – RbCoV/HKU14 (87.9)
ECoV R2.7	MF083115.1 – PHEV (91.4)

Figure S8. Recombination analysis of ECoV R2. A) IDPlot of ECoV Region 2 B-H) ML phylogenetic trees and distance tables for each putative sub-region of ECoV R2. I) BLAST analysis of ECoV R2 sub-regions.

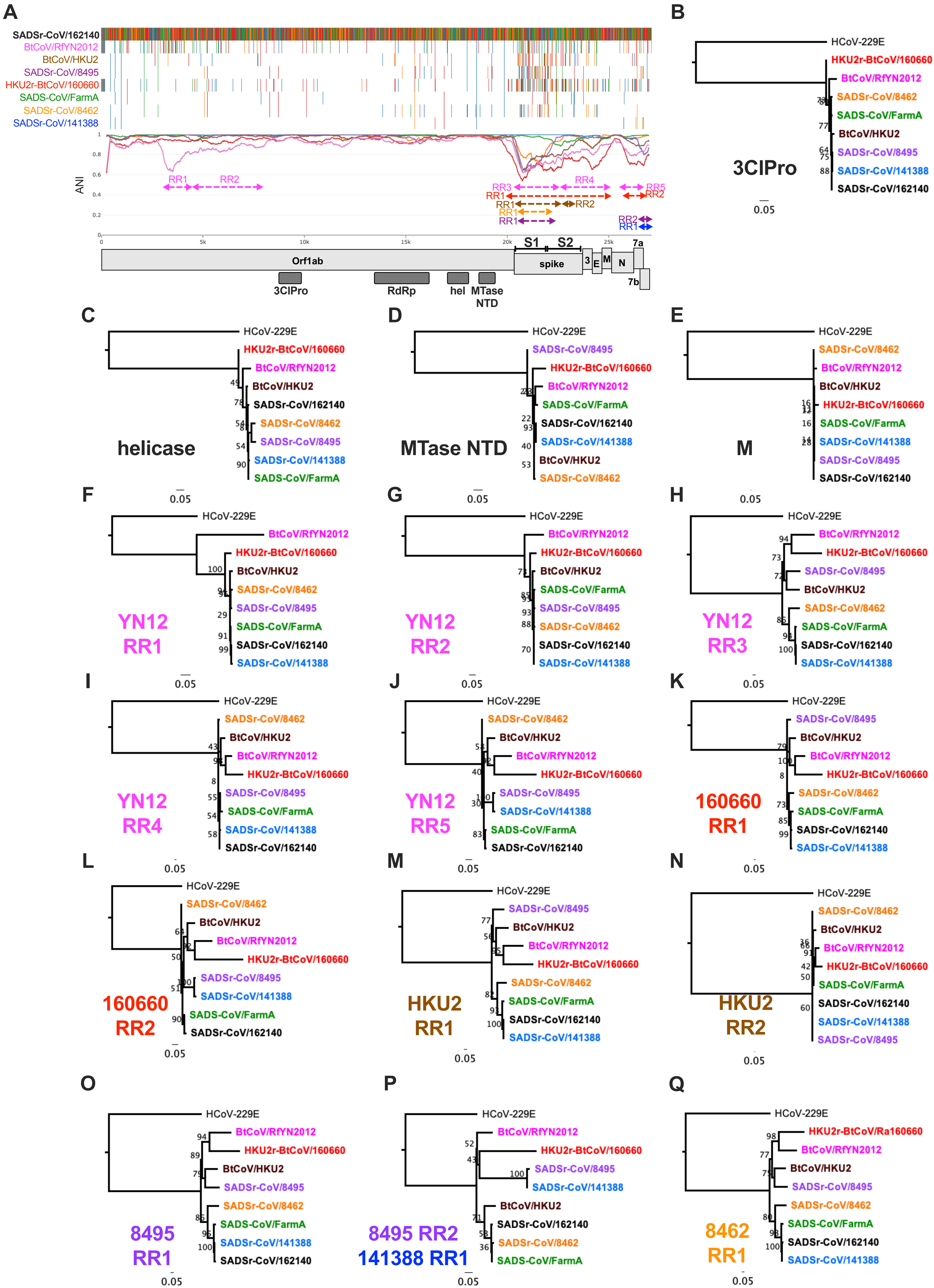

A
SADSr-CoV/162140
BtCoV/RfYN2012
BtCoV/HKU2
SADSr-CoV/8495
HKU2r-BtCoV/160660
SADS-CoV/FarmA
SADSr-CoV/8462
SADSr-CoV/141388
ANI
RR1
RR2
RR3
RR4
RR5
Orf1ab
spike
S1
S2
3ClPro
RdRp
hel
MTase NTD
B
3ClPro
C
helicase
D
MTase NTD
E
M
F
YN12 RR1
G
YN12 RR2
H
YN12 RR3
I
YN12 RR4
J
YN12 RR5
K
160660 RR1
L
160660 RR2
M
HKU2 RR1
N
HKU2 RR2
O
8495 RR1
P
8495 RR2
141388 RR1
Q
8462 RR1
HCoV-229E
HKU2r-BtCoV/Ra160660
0.05

Figure S9. Phylogenetic analysis of SADSr-CoV recombination. A) ANI plot of SADSr-CoVs including indication of recombinant regions identified by GARD and validated by our phylogenetic analysis. B-D) Maximum likelihood phylogenetic trees of conserved (non-recombinant origin) SADSr-CoV regions. MTase NTD is the furthest 3' region upstream of the first breakpoint surrounding SADSr-CoV spikes. M is downstream of several recombinant regions and upstream of recombinant regions encompassing 3' structural and accessory genes. E-Q) ML phylogenetic analysis of SADSr-CoV recombinant regions.

A

Region	Position (in native sequence)	% identity to reference	BLAST hits (% identity)
RfYN2012 RR1	2,854-3,657	67.5%	MF094687.1 – SADSr-CoV/141388 (69%)
RfYN2012 RR2	3,658-8,017	85.6%	MK994935.1 – SADS-CoV/GDWT-P18 (85.9%)
RfYN2012 RR3	20,287-22,002	67%	MF769424.1 – SADS-CoV/182 (69.1%)
RfYN2012 RR4	22,003-23,800	80.5%	EF203065.1 – BtCoV/HKU2-HK (81.3%)
RfYN2012 RR5	25,318-26,693	82.7%	MF094685.1 – SADSr-CoV/8462 (83.7%)
BtCoV/160660 RR1	19,807-24,631	69.5%	EF203065.1 – BtCoV/HKU2-HK (71.5%)
BtCoV/160660 RR2	25,547-26,738	71.9%	MF094688.1 – SADSr-CoV/162140 (71.9%
BtCoV/HKU2 RR1	20,489-22,496	73.9%	MF769407.1* - SADSr-CoV/141194 (99.8%)<br>EF203065.1 – BtCoV/HKU2-HK (98.8%)<br>MF769415.1* – SADSr-CoV/NL140388 (98.6%)<br>MF769414.1* – SADSr-CoV/140344 (96.4%)<br>MF094686.1 – SADSr-CoV/8495 (75.6%)
BtCoV/HKU2 RR2	22,497-23,252	86.2%	EF203067.1 – BtCoV/HKU2-HK (89.1%)<br>MF094688.1 – SADSr-CoV/162140 (86.2%)
SADSr-CoV/8462 RR1	20,530-22,044	77.3%	MF094700.1* – SADSr-CoV/HD13590 (99.9%)<br>MF094697.1* – SADSr-CoV/8355 (98.6%)<br>MF094691.1* – SADSr-CoV/8462 (96.8%)<br>MK977618.1 – SADS-CoV/p10 (78%)
SADSr-CoV/8495 RR1	20,501-22,167	71.4%	MF094689.1* – SADSr-CoV/8495 (84.5%)<br>EF203065.1 – BtCoV/HKU2-HK (75.2%)
SADSr-CoV/8495 RR2	26,653-26,942	70.7%	MF094687.1 – SADSr-CoV/141388 (98.6%)<br>MK994935.1 – SADS-CoV/GWDT-p18 (75.7%)
SADSr-CoV/141388 RR1	26,629-26,918	71.4%	MF094686.1 - SADSr-CoV/8495 (98.6%)<br>MK994935.1 – SADS-CoV/GWDT-p18 (74.8%)

B

MTase NTD

	162140	141388	FarmA	HKU2	8495	8462	BtCoV/16...	RfYN2012
162140		100%	97.7%	99.2%	96.7%	99.0%	93.6%	96.2%
141388	100%		97.7%	99.2%	96.7%	99.0%	93.6%	96.2%
FarmA	97.7%	97.7%		98.5%	95.7%	98.2%	93.6%	96.2%
HKU2	99.2%	99.2%	98.5%		96.9%	99.7%	94.4%	96.4%
8495	96.7%	96.7%	95.7%	96.9%		96.7%	93.1%	95.2%
8462	99.0%	99.0%	98.2%	99.7%	96.7%		94.1%	96.2%
BtCoV/160660	93.6%	93.6%	93.6%	94.4%	93.1%	94.1%		92.3%
RfYN2012	96.2%	96.2%	96.2%	96.4%	95.2%	96.2%	92.3%	

C

M

	162140	141388	FarmA	HKU2	8495	8462	BtCoV/16...	RfYN2012
162140		97.7%	97.7%	98.3%	98.1%	99.0%	89.7%	91.4%
141388	97.7%		98.8%	97.2%	97.4%	96.7%	90.1%	91.6%
FarmA	97.7%	98.8%		96.7%	97.4%	97.0%	90.1%	91.8%
HKU2	98.3%	97.2%	96.7%		97.7%	97.5%	89.1%	91.9%
8495	98.1%	97.4%	97.4%	97.7%		97.4%	89.0%	91.4%
8462	99.0%	96.7%	97.0%	97.5%	97.4%		89.4%	91.9%
BtCoV/160660	89.7%	90.1%	90.1%	89.1%	89.0%	89.4%		87.0%
RfYN2012	91.4%	91.6%	91.8%	91.9%	91.4%	91.9%	87.0%	

Figure S10. Breakpoint and nucleotide identity analysis of SADSr-CoV recombination. A) Breakpoint analysis of SADSr-CoVs including nucleotide identity to reference sequence and top BLAST hits. BLAST result accession numbers with an asterisk indicate the entry is a partial spike sequence only. B -C) Distance tables for MTase NTD, and M genes shows high nucleotide identity across SADSr-CoVs upstream and downstream of spike-encompassing recombinant regions.

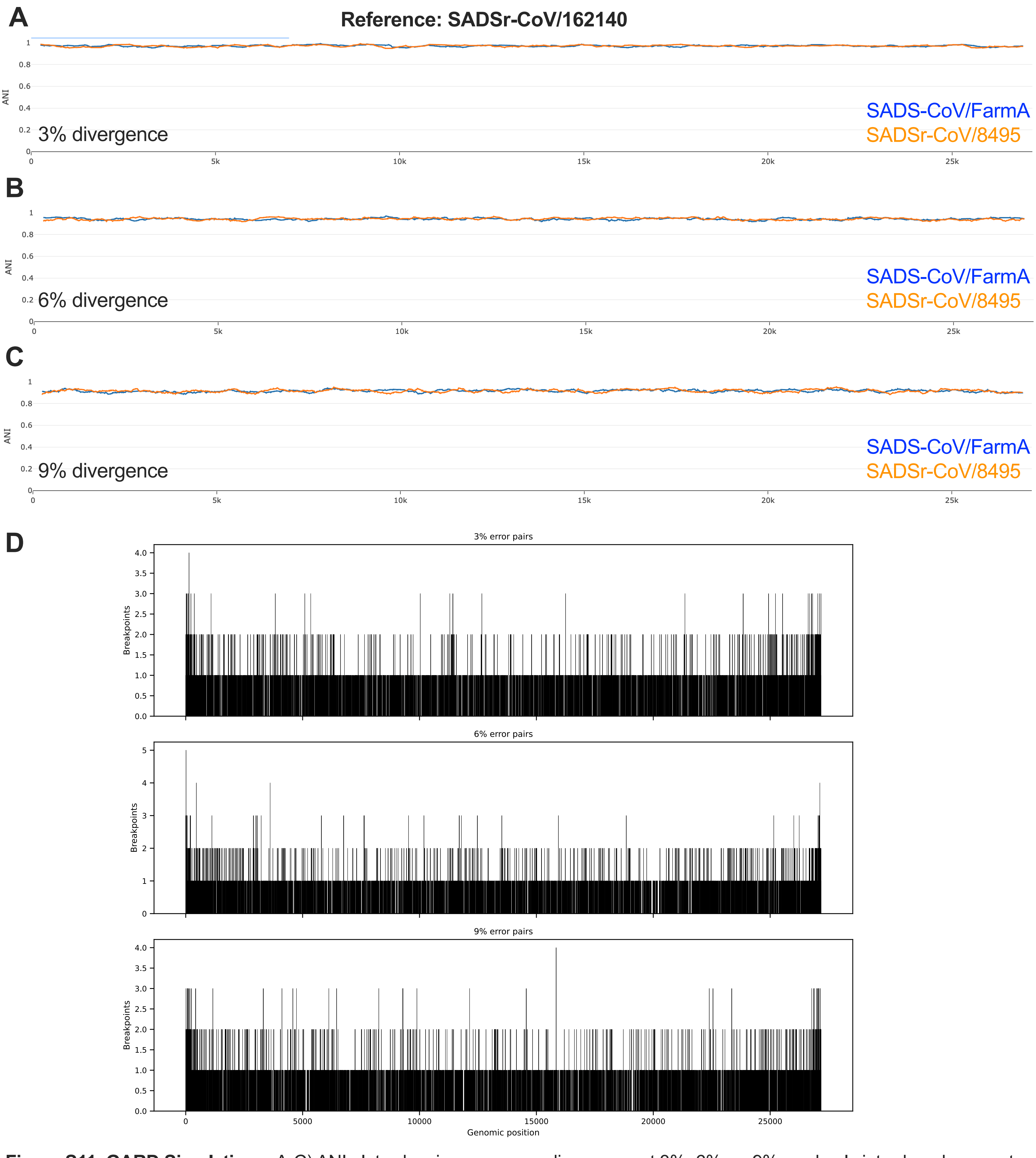

Figure S11. GARD Simulations. A-C) ANI plots showing sequence divergence at 3%, 6%, or 9% randomly introduced error rates D) Histograms showing even breakpoint distribution for each simulation.
